# Common polymorphisms in *GSTM1, GSTT1, GSTP1, GSTA1* and susceptibility to colorectal cancer in the Central European population

**DOI:** 10.1186/2047-783X-17-17

**Published:** 2012-06-14

**Authors:** Renata Hezova, Julie Bienertova-Vasku, Milana Sachlova, Veronika Brezkova, Anna Vasku, Marek Svoboda, Lenka Radová, Igor Kiss, Rostislav Vyzula, Ondrej Slaby

**Affiliations:** 1 Department of Comprehensive Cancer Care, Masaryk Memorial Cancer Institute, Zluty kopec 7, 656 53, Brno, Czech Republic; 2 Department of Pathological Physiology, Medical Faculty, Masaryk University, Kamenice 753/5, 625 00, Brno, Czech Republic; 3 Department of Paediatric Oncology, Masaryk University affiliated Hospital, Cernopolni 22, 612 00, Brno, Czech Republic; 4 Central European Institute of Technology, Masaryk University, Kamenice 753/5, 625 00, Brno, Czech Republic; 5 Laboratory of Experimental Medicine, Institute of Molecular and Translational Medicine, Faculty of Medicine and Dentistry, Palacky University and University Hospital in Olomouc, Krízkovského 8, 771 47, Olomouc, Czech Republic

**Keywords:** Colorectal cancer, *GSTA1*, *GSTT1*, *GSTM1*, *GSTP1*, Polymorphism

## Abstract

**Background:**

Central Europe presents with the highest incidence of sporadic colorectal cancer (CRC) worldwide. As sporadic CRC represents a typical multifactorial disease, it is characterized by intense interaction of the genetic background with the environment. Glutathione S-transferases could act as attractive susceptibility genes for CRC, as they are directly involved in conjugation between glutathione and chemotherapeutics, environmental pollutants and a wide spectrum of xenobiotics.

**Methods:**

In this study, we investigated associations of polymorphisms in glutathione S-transferases (*GSTs*) genes, that is *GSTA1*, *GSTT1*, *GSTM1* and *GSTP1*, with CRC in a total of 197 cases and 218 controls originating from the Czech Central European population. Polymorphisms were assessed by polymerase chain reaction/restriction fragment length polymorphism-based methods, allele-specific multiplex and allelic discrimination by real-time polymerase chain reaction.

**Results:**

None of investigated polymorphisms showed any associations with CRC, with the exception of *GSTP1;* where the heterozygote genotype Ile105Val was associated with decreased risk of CRC (*P* = 0.043).

**Conclusions:**

The frequencies observed in our study are in accordance with those from other European Caucasian populations. Based on our studies, examined variability in *GST* genes is not a major determinant of CRC susceptibility in the Central European population.

## Background

Colorectal cancer (CRC) represents the third most frequent type of cancer among males and the second most common cancer in females worldwide. Worldwide, every year, more than 1 million individuals will develop colorectal cancer and the disease-specific mortality rate is nearly 33% in the developed world [1], making the disease a substantial health as well as economic burden on society. Sporadic CRC is a typical multifactorial disease arising from maladaptive interaction between genetic background and certain environmental factors, such as diet or lifestyle, however, the exact role of the genetic background to sporadic CRC remains unclear.

Glutathione S-transferases (*GST*s) represent a superfamily of phase II metabolic enzymes that catalyze the conjugation between glutathione and chemotherapeutic drugs, carcinogens, environmental pollutants, and a broad spectrum of xenobiotics. *GST*s are involved in the metabolism of isothiacyanates (ITCs), naturally occurring molecules that were recently shown to inhibit development of tumors in many experimental models [2] and that also induce apoptosis in human colon cancer cells [3]. *GST* izoenzymes are encoded by three separate families of genes (designated cytosolic, microsomal and mitochondrial transferases), with distinct evolutionary origins, which provide mammalian species with protection against electrophiles and oxidative stressors in the environment. Members of the cytosolic class Alpha, Mu, Pi and Theta *GST*, and also certain microsomal transferases (MGST2 and MGST3), are upregulated by a diverse spectrum of foreign compounds typified by phenobarbital, 1,4-bis[2-(3,5-dichloropyridyloxy)] benzene, pregnenolone-16α-carbonitrile, 3-methylcholanthrene, 2,3,7,8-tetrachlorodibenzo-p-dioxin, β-naphthoflavone, butylated hydroxyanisole, ethoxyquin, oltipraz, fumaric acid, sulforaphane, coumarin, 1-[2-cyano-3,12-dioxooleana-1,9(11)-dien-28-oyl]imidazole,12-O-tetradecanoylphorbol-13-acetate, dexamethasone and thiazolidinediones.

The polymorphisms in these genes lead mostly to reduction of enzymatic activity, for example, homozygotes for polymorphisms C69T in *GSTA1* genes have reduced enzymatic activity compared to the wild-type homozygotes [4]. Also *GSTP1*, the most frequently distributed *GST* isoenzyme [5], harbors a functional polymorphism (rs1695, Ile105Val) [6] resulting in a lower enzyme activity [7]. Null genotype with no enzymatic activity in *GSTM1* gene was reported in 40 to 60% of Caucasians [8] and *GSTT1* null genotype is present at a frequency of 10 to 20% in Caucasian population [9]. Individuals with reduced GST enzymatic activity may present with decreased metabolism of ITCs, which could result in an increased pool of ITCs and, therefore, modified CRC risk.

The Central European population represents a population with extremely high incidence of CRC [10] and it could well serve as a model population for the study of the genetic background to CRC. The role of *GST* izoenzymes has been already studied in the Czech population of 495 CRC patients in a larger multicentric study. The aim of our study was to replicate these findings on a smaller, highly homogeneous cohort [11] of CRC patients, that is, to determine whether common genetic polymorphisms in the most important isoenzymes of the *GST, * family (*GSTA, GSTM1, GSTP1, * and *GSTT1)* predispose to the development of CRC cancer in this model population.

## Methods

### Patients and controls

*De novo* diagnosed cases of CRC treated at the Masaryk Memorial Cancer Institute, Czech Republic, between January 2008 and December 2010 were enrolled into this study. The cases comprised a total of 197 subjects [105 men, 92 women; age (mean ± SD): 63 ± 9 years) with the histologically confirmed diagnosis of CRC, whereas the control group included 218 cancer-free blood donor volunteers recruited from the same institute, had a similar age distribution (96 men, 122 women; mean age: 66 ± 16 years) and had no previous personal history of cancer. Colonoscopy was not performed in controls to exclude CRC, but all subjects were symptom-free and presented with no anemia. All subjects had the same ethnicity (Caucasian). Written informed consent approved by the local ethical review board was obtained from all subjects and was archived.

### DNA isolation and genotyping

Genomic DNA was isolated from whole peripheral blood using MagNA Pure DNA Isolation Kit (Roche Applied Science, Indianapolis, IN, USA). DNA concentration was measured on Nanodrop ND-1000 (NanoDrop Technologies Inc., Wilmington, DE, USA).

For analyses of SNPs rs1695 in *GSTP1* Ile105Val real-time PCR allelic discrimination was performed on a StepOne Real-Time PCR instrument (Life Technologies, Applied Biosystems, Carlsbad, CA, USA) using standard TaqMan genotyping assays according to the manufacturer’s instructions (Assay ID: C 3237198_20).

The analysis of the *GSTA1* C69T SNP was performed using PCR-restriction fragment length polymorphism (RFLP) according to [12]. A 400-bp fragment was amplified with the forward and reverse primers for *GSTA1* (F: 5′-GCATCAGCTTGCCCTTCA-3′; R: 5′-AAACGCTGTCACCGTCCTG-3′). PCR was performed with a total volume of 12.5 μl with 0.1 μg genomic DNA, 0.1 μM each primer, 0.2 mM each dNTP and 0.35 unit Taq DNA polymerase in 1x buffer supplied with the polymerase. Thermocycler parameters included an initial five-minute denaturation step at 94°C followed by thirty cycles of denaturation (94°C/20s), annealing (64°C/20s) and extension (72°C/30s). A final seven-minute extension was performed before cooling reaction to 4°C. All amplification steps were completed using PTC-200 Peltier thermal cycler (Bio-Rad Laboratories Inc, Hercules, CA USA). For RFLP analysis, 5 μl of PCR product was digested using 0.5 unit restriction enzyme Ear I with 1x buffer Tango (Fermentas Inc., Glen Burnie, MD, USA) supplied with restriction enzyme in total volume of 20 μl. Digestion was performed for 12 hours at 37°C; digestion was stopped by incubation at 65°C for 20 minutes. These digested products were separated on 2% agarose gel stained with ethidium bromide for 40 minutes. The wild-type allele (C) had no Ear I site and was still 400 bp. A 92-bp nucleotide was removed from the variant allele (T), which yielded in a 308-bp fragment.

Genotyping of *GSTM1* and *GSTT1* deletions was carried out using a duplex PCR with the *Albumin* gene serving as an internal positive control. The total reaction volume was 12.5 μl containing 0.1 μg genomic DNA, 0.35 units of Taq DNA polymerase (Fermentas Inc., Glen Burnie, MD, USA) in 1x Taq buffer supplied with polymerase, 0.1 μM each primer (GSTM1_F, GSTM1_R, ALB_F, ALB_R, or GSTT1_F, GSTT1_R, ALB_F, ALB_R) and 0.2 mM each dNTP. The sequence of reverse and forward primers were as follows:

 GSTM1_R5′-GAACTCCCTGAAAAGCTAAAGC-3′;

 GSTM1_F5′-GTTGGGCTCAAATATACGGTGG-3′;

 GSTT1_R5′-TCACCGGATCATGGCCAGCA-3′;

 GSTT1_F5′-TTCCTTACTGGTCCTCACATCTC-3′;

 ALB_R 5′-GCCCTAAAAAGAAAATCGCCAATC-3;

 ALB_F 5′-GCCCTCTGCTAACAAGTCCTAC-3′

All primers were synthesized at Integrated DNA Technologies Inc., Coralville, IA, USA. PCR conditions were the same as for the amplification of *GSTA1* mentioned above. The PCR products were analyzed on the 2% agarose gel stained with ethidium bromide for 40 minutes at 100 V. The wild-type genotype for *GSTM1* corresponded to 215 bp, *GSTT1* to 480 bp and the 380 bp corresponded to the *Albumin* gene, which was used as internal control to prove the successful PCR amplification [13,14].

### Statistical analysis

Results were evaluated in the Statistica version 9.0 program (StatSoft, Inc, Tulsa, OK, USA). Hardy-Weinberg equilibrium was tested for each polymorphism calculating *χ*^2^ test for the patients and the controls separately. When calculating odds ratio (OR), the homozygote of the most frequent allele was used as a reference. For odds ratio and 95% confidence interval, unconditional logistic regression was used based on a model adjusted for sex and age of patients. The values of *P* < 0.05 were considered statistically significant.

## Results

All investigated polymorphisms were in Hardy-Weinberg equilibrium. The frequencies of *GST* genotypes by case-control status and the association of *GST* polymorphisms with CRC are shown in Table 1. An unconditional logistic regression model was employed to compare the genotype and allele frequencies of SNPs in *GSTP1* (rs1695, Ile105Val), *GSTA1* (C69T substitution) and deletion in *GSTM1* and *GSTT1* genes between the cases and the controls. There were no significant differences in allele and genotype distributions between the investigated cohorts. A logistic regression model was used to identify the OR of the CRC of investigated genetic variants. The subjects carrying heterozygous genotype of *GSTP* rs1695: A > G had significantly decreased risk of CRC (OR 0.64; 95% CI: 0.42 to 0.98, *P* = 0.043) compared to AA homozygotes. The frequency of AG heterozygotes of *GSTP1* rs1695 was lower in CRC patients than in healthy controls (37.56% versus 45.87%), G allele-containing genotypes (AG and GG) showed a similar tendency in their frequencies (28.93% in CRC versus 34.40% in controls). The investigated polymorphisms in *GSTA1* as well as *GSTM1* and *GSTT1* genes failed to show a statistically significant association with CRC risk.

**Table 1:** **Logistic regression analyses of genotype frequencies of SNPs *GSTP1 *(rs1695), *GSTA1 *and deletion in *GSTM1 *and *GSTT1 *in colorectal cancer cases and controls from a Czech population**

		**Control**		**CRC**				
		**N**	**%**	**N**	**%**	OR***	**95% Cl**	***P-value***
***GSTA1*(C69T)**	CC	76	34.8	69	35,0	1.00		0.981*
	CT	108	49.5	99	50.3	1.04	(0.67–1.60)	0.847**
	TT	34	15.6	29	14.7	1.03	(0.56–1.90)	0.960**
	Trend	218		197		1.02	(0.76–1.37)	0.887*
***GSTT1*_del**	wt	179	82.1	157	79.7	1.00		0.531*
	del	39	17.9	40	20.3	1.13	(0.69–1.86)	0.711**
	Trend	218		197		1.13	(0.69–1.86)	0.531*
***GSTM1*_del**	wt	117	53.7	97	49.2	1.00		0.262*
	del	101	46.3	100	50.7	1.25	(0.84–1.86)	0.276**
	Trend	218		197		1.25	(0.84–1.86)	0.263*
***GSTP1*(Ile105Val,rs1695)**	AA	93	42.6	103	52.3	1		0.095*
	**AG**	**100**	**45.9**	**74**	**37.6**	**0.64**	**(0.42**–**0.98)**	0.043**
	GG	25	11.5	20	10.2	0.67	(0.35–1.28)	0.266**
	Trend	218		197		0.76	(0.56–1.02)	0.062*

*LR-test.

**Wald’s test.

***Age and sex adjusted.

## Discussion

In this study, we investigated the possible role of common *GST* polymorphisms in the development of CRC in the Central European Caucasian population, a population with the highest incidence of CRC worldwide. The reports on *GST* polymorphisms as potential risk factors for CRC are controversial [15]. Based on this large meta-analysis of SNPs in *GSTM1, GSTT1, GSTP1, * and *GSTA1, * genes, the *GSTM1* as well as *GSTT1* null allele carriers should exhibit increased CRC risk in Caucasian populations.

However, the only significant association from our study refers to the *GSTP1* Ile105Val polymorphism, for which no significant associations were reported in large pooled Chinese or Caucasian cohorts [15], with an exception of a very small Bulgarian population [16]. Surprisingly, the frequencies of *GSTP1* genotypes observed in our study were significantly different between our study and another study on a Czech population mentioned above [11] (52.2% vs. 45.1%) and the frequency of heterozygotes was also markedly different between these two cohorts (37.6% vs. 46.3%).

On the whole, the frequencies of examined polymorphism in our study corresponded well with other European cohorts. The frequencies of *GSTA1* polymorphism in our population were similar to the frequencies in a Dutch cohort [17], frequencies of *GSTT1* genotypes were slightly below the frequencies reported for the Scottish [18] population. The frequencies of *GSTM1* genotypes also corresponded well to the Dutch [17] and Scottish cohort [18]. As for the genotype frequencies of rs1695 in *GSTP1* gene, the AA genotype was significantly more frequent in our cohort than in the Dutch study (52.2% vs. 42.5%), however, less frequent than in the Bulgarian study [16] (52.2% vs. 69.0%).

When comparing the frequency of *GSTM1* null allele in CRC cases between our study and the study by Hlavata *et al*. [11], significant differences were observed for the *GSTM1* null allele (53.9% vs. 50.8%), while frequency of *GSTT1* null allele was almost identical between our study and their study (20.8% vs. 20.3%).

As mentioned above, in this study we observed a significantly decreased CRC risk associated with heterozygosity in *GSTP1* locus. The biological relevance of this is unclear. It has to be mentioned that the low-expression variant (GG) of this polymorphism is expressed in a recessive manner, thus both alleles have to be present to express the trait. The possible explanation for the protective effect associated with heterozygosity in *GSPT1* locus could be an unknown advantage of the heterozygotes related to yet undiscovered mechanisms of protection against sporadic CRC. In the above mentioned study by Hlavata *et al*. [11], no such association was observed for Ile105Val polymorphism in *GSTP1* locus (OR 1.01, 95%CI, 0.77 to 1.32, *P* = 0.944), but a similar trend for the *GSTM1* deletion and its association with CRC risk was observed (OR 1.30, 95% CI 1.01 to 1.68, *P* = 0.044), which can be easily explained by the similar ethnic origin of both the cohorts [11].

Limitations of the study include a significant lack of data on phenotypic effects of *GST* family genes, which makes further functional studies a necessity to determine the exact genotype-phenotype correlation, with special attention paid to the dietary composition, mainly in terms of the amount of isothiocyanates in the diet. It may be suggested that our results are affected by selection bias, however, the patients and the controls were included in the study randomly, as they arrived at the clinic. Also, it should be mentioned that the present study was carried out on a relatively static population from Moravia, part of the Czech Republic settled by a Central European Caucasian population of Slavonic origin that can be assumed to be homogeneous, and that was previously associated with extreme rates of CRC compared to other populations.

## Conclusions

Frequencies of the polymorphisms in *GST* genes observed in our study are in accordance with those from other European Caucasian populations. Our data do not provide a robust evidence that investigated polymorphisms in *GST* genes act as major modulators of genetic susceptibility to CRC in the Central European population.

## Competing interests

The authors declare that they have no competing interests.

## Authors’ contributions

RH and AK performed the DNA purifications and SNP analysis. MS, IK, MS, AV, VB and RV collected the DNA samples and clinical data of patients and controls involved in the study. LR performed statistical evaluation of the data. OS, RH and JBV participated in the manuscript preparation. OS and JBV designed the study, performed analysis and interpretation of data, and critical revision of the manuscript. All authors read and approved the final manuscript.
